# Parameters Governing the Community Structure and Element Turnover in Kermadec Volcanic Ash and Hydrothermal Fluids as Monitored by Inorganic Electron Donor Consumption, Autotrophic CO_2_ Fixation and 16S Tags of the Transcriptome in Incubation Experiments

**DOI:** 10.3389/fmicb.2019.02296

**Published:** 2019-10-09

**Authors:** Stefanie Böhnke, Katharina Sass, Giorgio Gonnella, Alexander Diehl, Charlotte Kleint, Wolfgang Bach, Rebecca Zitoun, Andrea Koschinsky, Daniela Indenbirken, Sylvia G. Sander, Stefan Kurtz, Mirjam Perner

**Affiliations:** ^1^Molecular Biology of Microbial Consortia, Institute of Plant Science and Microbiology, Universität Hamburg, Hamburg, Germany; ^2^Center for Bioinformatics (ZBH), Universität Hamburg, Hamburg, Germany; ^3^Department of Geosciences, MARUM – Centre for Marine Environmental Sciences, University of Bremen, Bremen, Germany; ^4^Department of Physics and Earth Sciences, Jacobs University Bremen, Bremen, Germany; ^5^Department of Chemistry, University of Otago, Dunedin, New Zealand; ^6^Heinrich Pette Institute, Leibniz Institute for Experimental Virology, Hamburg, Germany

**Keywords:** microbial hydrogen oxidation, microbial iron oxidation, microbial sulfide oxidation, autotrophic CO_2_ fixation, microbial hydrothermal vent communities, MiSeq, 16S rRNA genes

## Abstract

The microbial community composition and its functionality was assessed for hydrothermal fluids and volcanic ash sediments from Haungaroa and hydrothermal fluids from the Brothers volcano in the Kermadec island arc (New Zealand). The Haungaroa volcanic ash sediments were dominated by epsilonproteobacterial *Sulfurovum* sp. Ratios of electron donor consumption to CO_2_ fixation from respective sediment incubations indicated that sulfide oxidation appeared to fuel autotrophic CO_2_ fixation, coinciding with thermodynamic estimates predicting sulfide oxidation as the major energy source in the environment. Transcript analyses with the sulfide-supplemented sediment slurries demonstrated that *Sulfurovum* prevailed in the experiments as well. Hence, our sediment incubations appeared to simulate environmental conditions well suggesting that sulfide oxidation catalyzed by *Sulfurovum* members drive biomass synthesis in the volcanic ash sediments. For the Haungaroa fluids no inorganic electron donor and responsible microorganisms could be identified that clearly stimulated autotrophic CO_2_ fixation. In the Brothers hydrothermal fluids *Sulfurimonas* (49%) and *Hydrogenovibrio/Thiomicrospira* (15%) species prevailed. Respective fluid incubations exhibited highest autotrophic CO_2_ fixation if supplemented with iron(II) or hydrogen. Likewise catabolic energy calculations predicted primarily iron(II) but also hydrogen oxidation as major energy sources in the natural fluids. According to transcript analyses with material from the incubation experiments *Thiomicrospira/Hydrogenovibrio* species dominated, outcompeting *Sulfurimonas*. Given that experimental conditions likely only simulated environmental conditions that cause *Thiomicrospira/Hydrogenovibrio* but not *Sulfurimonas* to thrive, it remains unclear which environmental parameters determine *Sulfurimonas’* dominance in the Brothers natural hydrothermal fluids.

## Introduction

Hydrothermal fluids emitted from cracks and fissures of the seafloor transport numerous reduced chemical compounds to the surface. As the hot liquids ascend, they can mix with entrained cold, oxygenated seawater causing thermal and chemical gradients in the (sub)surface venting regions. Chemosynthetic microorganisms can exploit this thermodynamic disequilibrium gaining energy through catalyzing redox processes. The energy can be used to fix CO_2_ autotrophically and synthesize biomass.

Hydrothermal vents have been mostly studied along mid-ocean ridge (MOR) systems. However, those located in volcanic arc systems may be more important for global element fluxes than those from MOR systems because they are usually in much shallower waters (<1800 m, with more than 50% of the vent sites in depths <500 m) (de Ronde et al., 2003). Hydrothermal fluids emitting from island arcs exhibit a higher variability regarding their chemical compositions, due to a larger range in water depth, higher variability in host rock composition as well as magmatic input (e.g., German et al., 2006; Craddock et al., 2010; de Ronde et al., 2011). At Brothers Volcano, endmember fluids showing intense water/rock interaction (along the NW caldera wall) as well as fluids showing strong magmatic volatile input (at the two cones in the SE) have been observed and sampled in the same caldera, while fluids at Haungaroa are only dominated by water/rock interactions (Kleint et al., 2019). Only a few studies on microbial community compositions from the Kermadec island arc system exist: at the Brothers volcano Archaea and Bacteria were quantified in chimneys (Takai et al., 2009) and the microbial diversity was assessed from a rock at a venting outlet based on 16S rRNA and functional genes (Stott et al., 2008). Their findings indicated a pronounced role for microbially catalyzed hydrogen oxidation, reduction of sulfate and other sulfur compounds at these vent sites (Stott et al., 2008; Takai et al., 2009). Additionally, iron-oxidizing *Zetaproteobacteria* were identified in iron-flocks and sediments at the southern-most Kermadec arc locations (Hodges and Olson, 2009). Here, particularly the dominance of phylogenetically diverse uncultured *Epsilonproteobacteria* was obvious in flocculent mats (Hodges and Olson, 2009). Members of the *Epsilonproteobacteria* are generally very common inhabitants of geographically and chemically distinct hydrothermally influenced environments (for review see Campbell et al., 2006). They are known for their metabolic versatility (e.g., hydrogen or sulfide oxidation) and have a major impact on local element cycling (e.g., sulfur and nitrogen cycling, Fe-S-mineral precipitation, the use of iron(III) as electron acceptor) (Campbell et al., 2006).

Microbial communities colonizing sediments (even with little hydrothermal input) are considerably different to those that are found in hydrothermal fluids (Cerqueira et al., 2015, 2017). Volcanic ash as we find in the Kermadec arc, is a special type of sediment which is created during explosive volcanic eruptions. Here, large quantities of ash particles are expelled, spread over a wide area and deposited at the seafloor (Witt et al., 2017). It consists of pulverized rock, minerals and volcanic glass fragments. Work on marine microbial communities found in volcanic ash is very limited (Inagaki et al., 2003a; Witt et al., 2017). Volcanic ash marine microbial communities have been associated with members of the *Alpha*- and *Gammaproteobacteria* as well as affiliates of the Miscellaneous Crenarchaeotic Group (MCG) (Inagaki et al., 2003a).

Here we compare the microbial community compositions and their functionality in two hydrothermal fluid and one tephra (volcanic ash sediment) samples from two locations along the Kermadec island arc system. For this purpose, incubation experiments were set up, consumption of amended inorganic electron donor and autotrophic CO_2_ fixation was measured. Additionally, the phylogenetic diversity of the transcriptomes for each type of incubation experiment was compared with each other and with the initial starting community composition.

## Results and Discussion

**FIGURE 1:**
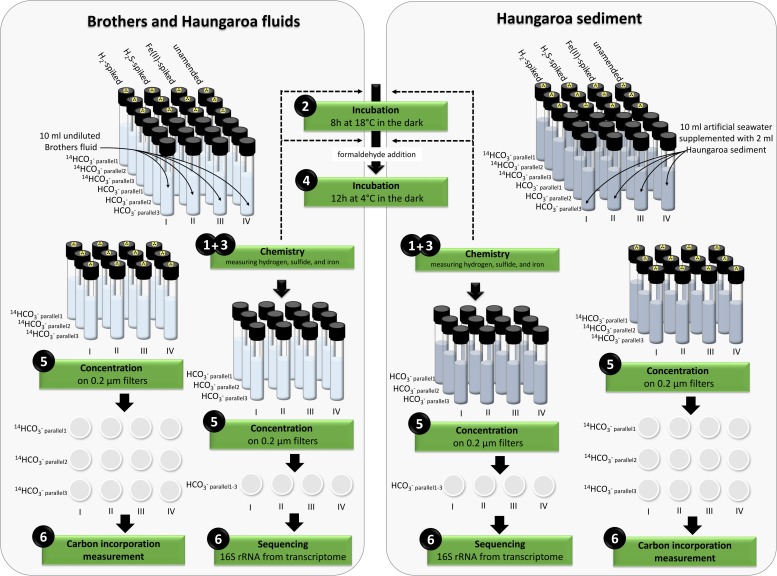
Overview of the set-up of the incubation experiments.

In the current study we investigated the composition and functionality of microbial communities inhabiting two distinct Kermadec island arc locations. These were (i) Haungaroa fluids (30 ROV 4F) and tephra (35 ROV 15M) and (ii) hydrothermal fluids from the Brothers volcano (61 ROV 13F–15F). Incubation experiments with fluid and tephra samples were conducted to identify whether hydrogen, iron(II) or sulfide (as most abundant inorganic electron donors) primarily fuel autotrophic CO_2_ fixation in these habitat types. Additionally, the active community of each type of incubation experiment was monitored by analyzing 16S rRNA gene tags from the transcriptome (for experimental set-up see Figure 1).

### Environmental Characteristics

**FIGURE 2:**
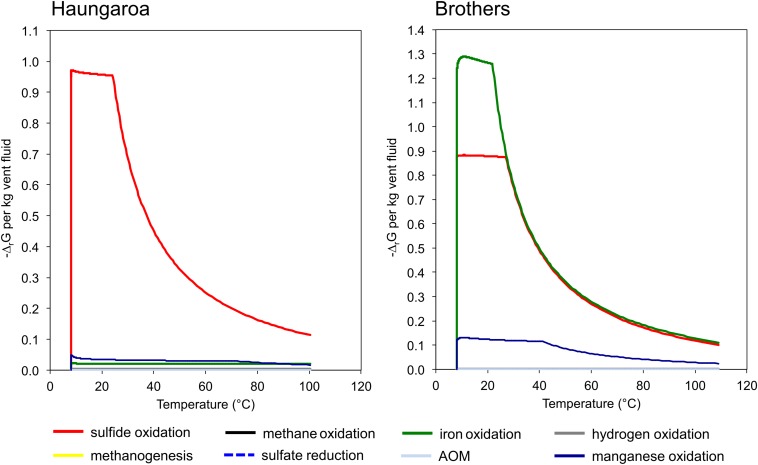
Quantities of Gibbs energy of varied catabolic reactions at Haungaroa and at Brothers NW caldera wall. Note that sulfide oxidation is predicted to constitute the dominant energy source at Haungaroa, whereas the oxidation of iron and also of sulfide dominate the potential catabolic energy at Brothers. See Amend et al. (2011) for computational details.

The sediment sample (35 ROV 15M) was collected from the Haungaroa area (∼677 m water depth) (Supplementary Figure 1), roughly 400 m away from the direct impact of hydrothermal fluids (30 ROV 4F) emitting from the subsurface. Since no geochemical data is available for porewater of these fluids we refer to hydrothermal emissions nearby to disclose general trends in chemical compositions. The highest *in situ* temperature for Haungaroa focused hydrothermal fluids was 267°C, iron(II) concentrations were up to 0.4 mM and sulfide levels reached up to 1.57 mM at a pH of 3.4 (Kleint et al., 2019). In these fluids, sulfide oxidation was estimated to be the major source of metabolic energy (Figure 2). Measured hydrogen in diffuse fluids was below 10 nM (W. Bach and A. Diehl, University Bremen, unpublished data). The actual measurements of the Haungaroa diffuse fluid samples collected for our experiments (30 ROV 4F) are outlined in Table 1.

**TABLE 1:** Compositions of diffuse fluids from two diffuse vent sites in the southern Kermadec arc and corresponding Gibbs energies for potential catabolic reactions.

							
							
	**Concentrations^∗^**	**Activities^#^**		**Catabolic reaction**	**Δ_r_G^0^ (kJ/mol)^$^**	**Δ_r_G (kJ/mol)^++^**	**Δ_r_G (kJ/mol e^–^)**
**Haungaroa (30 ROV 4F)**							
H_2_(aq)	0.008	0.008	μM	H_2_(aq) + 0.5O_2_(aq) = H_2_O	–527.56	–459.54	–229.27
CH_4_(aq)	0.02	0.02	μM	CH_4_(aq) + 2O_2_(aq) = HCO_3_**^–^** + H^+^ + H_2_O	–824.83	–819.37	–102.42
HCO_3_**^–^**	2.38	1.42	mM	H_2_S(aq) + 2O_2_(aq) = SO_4_^2^**^–^** + 2H^+^	–753.45		
H_2_S(aq)	bdl		μM	Fe^2+^ + 0.25O_2_(aq) + 2.5H_2_O = Fe(OH)_3_(s) + 2H^+^	–45.34		
Fe^2+^	bdl		μM	HCO_3_**^–^** + H^+^ + 4H_2_(aq) = CH_4_(aq) + 3H_2_O	–230.62	–177.75	–22.22
O_2_(aq)	155	155	μM	SO_4_^2^**^–^** + 2H^+^ + 4H_2_(aq) = H_2_S(aq) + 4H_2_O	–302.01		
SO_4_^2^**^–^**	28	2.7	mM				
**Brothers. NW caldera wall (61 ROV 13F–15F)**							
H_2_(aq)	0.126	0.126	μM	H_2_(aq) + 0.5O_2_(aq) = H_2_O	–527.34	–466.31	–233.16
CH_4_(aq)	0.092	0.092	μM	CH_4_(aq) + 2O_2_(aq) = HCO_3_**^–^** + H^+^ + H_2_O	–824.47	–818.12	–102.26
HCO_3_**^–^**	2.87	1.71	mM	H_2_S(aq) + 2O_2_(aq) = SO_4_^2^**^–^** + 2H^+^	–752.55	–794.01	–99.25
H_2_S(aq)	15	15	μM	Fe^2+^ + 0.25O_2_(aq) + 2.5H_2_O = Fe(OH)_3_(s) + 2H^+^	–45.45	–69.93	–69.93
Fe^2+^	179	42.7	μM	HCO_3_**^–^** + H^+^ + 4H_2_(aq) = CH_4_(aq) + 3H_2_O	–230.56	–182.66	–22.83
O_2_(aq)	229	229	μM	SO_4_^2^**^–^** + 2H^+^ + 4H_2_(aq) = H_2_S(aq) + 4H_2_O	–302.50	–206.79	–25.85
SO_4_^2^**^–^**	28	2.7	mM				

*^∗^Data are mostly from Kleint et al. (2019); some H_2_S (total sulfide) and HCO_3_**^–^** (DIC) are unpublished data, H. Strauss; H_2_ and CH_4_ are unpublished data, W. Bach, Univeristy of Bremen. ^#^Unity activity coefficients were assumed for neutral molecules; those for other solutes were computed using the extended Debye–Hückel equation. ^$^Standard Gibbs energy for ambient pressure and temperature. ^++^Actual Gibbs energy mol of reaction. Haungaroa: T 18°C; pH 7.38; Brothers: T 21°C; pH 6.48.*

Collected Haungaroa sediments were covered with a white microbial mat (Supplementary Figure 2). Mussel patches nearby suggest a source of diffuse fluids from below. The Haungaroa sediment sample is composed of poorly sorted tephra consisting of angular fragments of ash with minor lapilli (for chemical composition see Table 2). Three distinct rock types could be identified in the tephra. The most abundant type was plagioclase-phyric volcanic glass. Less abundant were dark-gray, microcrystalline plagioclase-phyric, vesicular fragments, in which the vesicles are commonly filled with secondary minerals. The least abundant type was white to cream-colored, well-rounded plagioclase-phyric pumice particles. Based on the bulk composition in a total alkali – silica diagram the volcanogenic sediment was classified as dacitic. This composition was distinctly different from that of fresh volcanic ash at Haungaroa, which are uniformly basaltic andesites (*n* = 5) (Diehl, 2019). The abundant vesicle infill as well as relatively high sulfur (2 wt. %), strontium (405 ppm) and barium concentrations (499 ppm) suggest that the sediment has been incipiently altered by hydrothermal fluids (Table 2). Since the sample was collected with a shovel and not via coring no porewater analyses exists for this site.

**TABLE 2:** Chemical analyses of tephra (volcanic ash sediment) from Haungaroa (035 ROV 15M).

**Compound**	**Concentration(wt. %)**	**Element**	**Concentration (mg kg^–1^)**
SiO_2_	68.3	As	18
TiO_2_	1.4	Ba	405
Al_2_O_3_	13.0	Co	18
Fe_2_O_3__tot_	6.3	Cr	21^∗^
MnO	0.1	Cu	136
MgO	1.7	Ga	15
CaO	3.6	Mo	6
Na_2_O	2.7	Nb	1^∗^
K_2_O	0.8	Ni	3^∗^
P_2_O_5_	0.2	Pb	15
Total S	2.0	Rb	11
Original totals	^§^ 92.0	Sr	499
		Th	0
		U	20
		V	285
		Y	2
		Zn	72
		Zr	71

*^∗^Below limit of detection. ^§^ Major components were normalized to 100%.*

Brothers Volcano (Supplementary Figure 1) has a ∼3 km wide caldera with active hydrothermal venting at the northwestern caldera wall and at two resurgent cones located in the southern part of the caldera. Fluid sample 061 ROV 13F–15F was collected from a diffuse venting site at the NW caldera wall in 1643 m water depth. The vent site showed issuing of shimmering water and there were abundant bacterial mats. The measured *in situ* temperature of the vent fluid ranged between 13 and 21°C during sample collection. Compared to the surrounding seawater, the vent fluids were enriched with iron(II) (179 μM) and sulfide (15 μM); the pH was 6.5 (Table 1). Hydrogen concentrations measured on board were 126 nM (W. Bach, University of Bremen, unpublished data).

The diffuse fluids likely formed during mixing of upwelling hydrothermal fluids and cold seawater entrained in the subseafloor. The endmember fluid composition, represented by 310°C hot black smoker fluids from Brothers NW caldera (Kleint et al., 2019) was also used to calculate the catabolic energy distribution upon mixing with seawater. The result of this computation shows that the oxidation of iron and sulfide likely constitutes the prevailing energy source for microbial metabolisms (Figure 2). The predicted compositions for the diffuse fluids from simple mixing of endmember hydrothermal fluids and seawater are presented in Supplementary Table 1. Predicted concentrations are close to the observed ones for methane and dissolved inorganic carbon (DIC). Sulfide, iron(II), and (to a lesser extent) hydrogen are depleted in the fluids relative to the predicted values, which indicates subseafloor loss of these components potentially due to microbial utilization or precipitation. In the Haungaroa fluids all components are depleted relative to the values from simple mixing of endmember hydrothermal vent fluid and seawater (Supplementary Table 1). It is hence possible that the actual contribution of the hydrothermal fluids may be smaller than indicated by the elevated temperature. Regardless, the depletion in sulfide is most pronounced indicating that the sulfide utilization predicted from the model (Figure 2) may indeed take place also at Haungaroa.

We also computed the Gibbs energies of a number of catabolic reactions for the diffuse fluids venting at Haungaroa and Brothers. This was achieved by using the measured composition of the fluids to calculate activities of the relevant dissolved species. These are then used to determine Δ_r_G from Δ_r_G^0^. The latter was computed for ambient pressure and temperature conditions using the program SUPCRT92 (cf. Amend et al., 2011). The results are listed in Table 1 and show that in Haungaroa and Brothers fluids most energy is available from hydrogen oxidation, while catabolic energy yields are lower for sulfide oxidation than those for hydrogenotrophy in both fluids. These results appear to be in contrast to the results of the catabolic energy calculations presented in Figure 2 that identify sulfide oxidation as a major energy source. This apparent contrast is due to the fact that sulfide is strongly depleted in the actual diffuse fluids when compared to the model mixed fluids (Supplementary Table 1). Methane, on the other hand, is not depleted. This data is most plausibly explained by massive sulfide loss from mineral precipitation and microbial utilization. Methane, in contrast, is apparently not consumed in the environment. These trends are most obvious in the diffuse fluids at Brothers. There, high iron contents predicted in the mixing calculations (Figure 2) lead to high energy yields for iron oxidation. The actual fluids have much lower iron contents than predicted from simple mixing, and, again, it is likely that subseafloor precipitation of iron led to this depletion. However, this depletion may also point toward microbial utilization of iron(II) as electron donor.

### Microbial Community Compositions in the Environments

**FIGURE 3:**
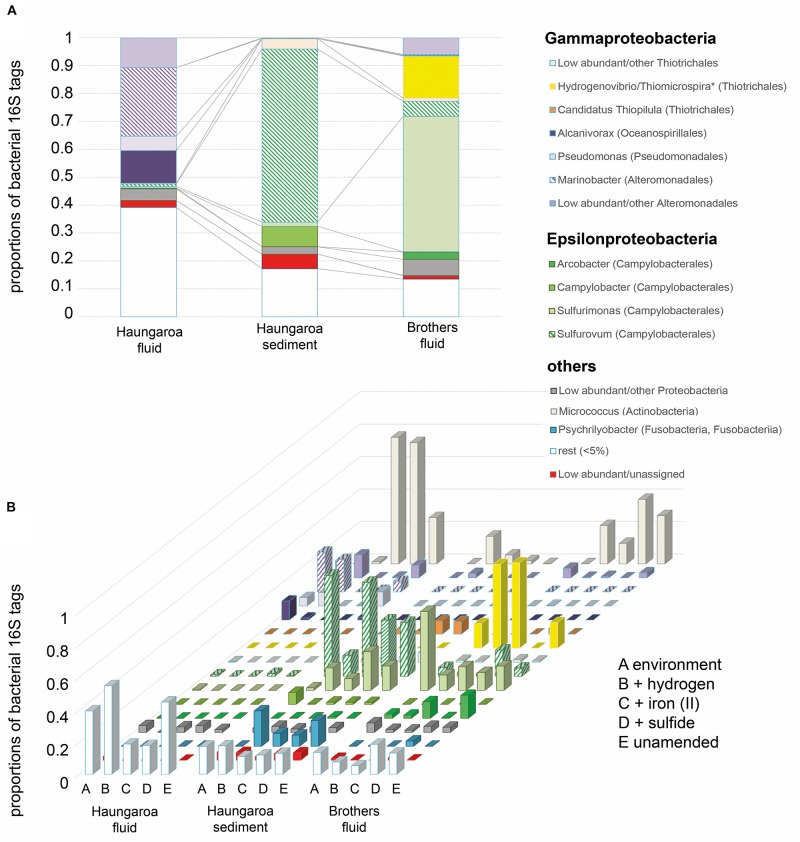
Bacterial 16S rRNA tags with **(A)** DNA extracted from the environment, i.e., the Haungaroa fluids (30 ROV 4F) and sediment sample (35 ROV 15M) and from the Brothers fluid sample (61 ROV 13F-15F) and **(B)** RNA isolated from incubation experiments with sediment and fluid material. ^∗^The genera *Thiomicrospira* and *Hydrogenovibrio* were recently evaluated and reclassified by Boden et al. (2017).

**FIGURE 4:**
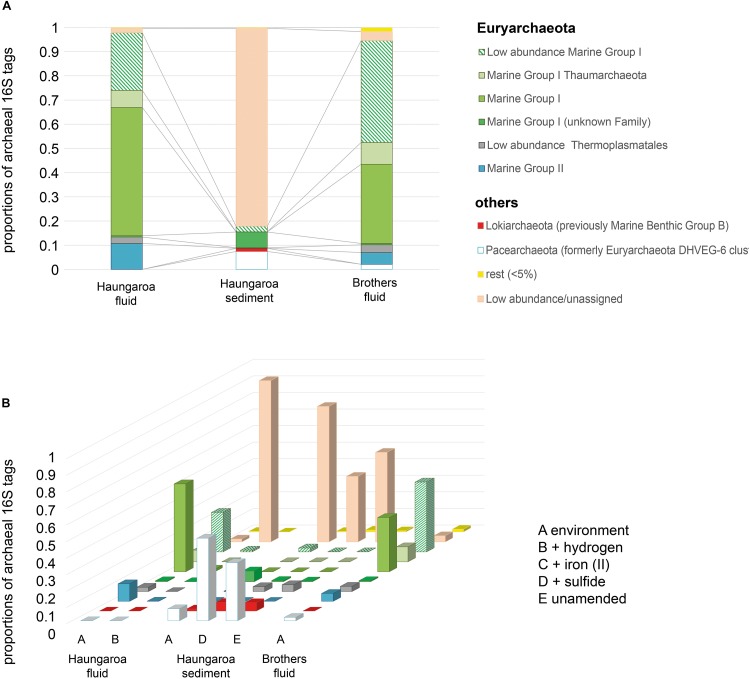
Archaeal 16S rRNA tags with **(A)** DNA extracted from the environment, i.e., Haungaroa fluids (30 ROV 4F) and sediment sample (35 ROV 15M) and from the Brothers fluid sample (61 ROV 13F-15F) and **(B)** RNA isolated from incubation experiments with sediment and fluid material.

The Haungaroa fluids and volcanic ash sediment were considerably different in their phylogenetic diversity (Figure 3A). The Haungaroa fluids contained nearly 40% of bacterial 16S rRNA gene tags which were present at low levels (i.e., <5%) suggesting a high phylogenetic diversity. The rest of the 16S rRNA gene tags were mostly dominated by *Gammaproteobacteria*, namely *Pseudomonas*, *Alcanivorax* as well as *Marinobacter* and other *Alteromonadales*. These microorganisms are organotrophs associated with moderate temperatures and oxygenated conditions (Gonzalesz and Whitman, 2006) but have been detected in fluids before (Perner et al., 2013a). Members of these genera are not commonly found in hydrothermally influenced habitats and their presence is likely due to entrainment of seawater. The archaeal community of the Haungaroa fluids was very similar to the composition detected in the Brothers fluids primarily related to affiliates of the Marine Group I Thaumarchaeota (Figure 4A) and could suggest entrainment from ambient seawater and surrounding sediments. *Thaumarchaeota* have been identified in a broad spectrum of organic-rich to organic-lean sediments (Durbin and Teske, 2012), which probably explains their ubiquitous distribution in marine benthic habitats.

The Haungaroa sediments were primarily dominated by *Epsilonproteobacteria*, namely *Sulfurovum* (62%) and *Campylobacter* (7%) species (Figure 3A). Previous studies of volcanic ash marine microbial communities have not identified *Epsilonproteobacteria* but members of the *Alpha*- and *Gammaproteobacteria* instead (Inagaki et al., 2003a). However, those samples were from depth up to 52.1 mbsf from a mix of (hemi-)pelagic clay and volcanic ash layers in the Sea of Okhotsk. Our sample, however, was from the upper volcanic ash sediment layer and appeared to be exposed to hydrothermal venting as indicated by a microbial mat (see Supplementary Figure 2), mussel patches nearby and sediment chemistry (Table 2). Among the Archaea low abundance and unassigned groups make up the majority of 16S rRNA gene tags (83%) (Figure 4A). Some Marine Group I *Thaumarchaeota* and the *Pacearchaeota* (formerly *Euryarchaeota* DHVEG-6 cluster) (Ortiz-Alvarez and Casamayor, 2016) constituted 7 and 5%, respectively. While originally detected in anoxic samples the *Pacearchaeota* have now also been recovered from surface waters indicating that they can cope with oxygen (Adam et al., 2017).

The bacterial community in the Brothers fluid was also dominated by *Epsilonproteobacteria*, but different genera, and some members of the *Gammaproteobacteria*. These were mostly *Sulfurimonas* (49%) and *Hydrogenovibrio*/*Thiomicrospira* (15%) species (recently evaluated and reclassified by Boden et al., 2017) (Figure 3A). Both *Sulfurimonas* and *Thiomicrospira/Hydrogenovibrio* have been frequently recognized in geographically and chemically distinct hydrothermal fluids (Perner et al., 2011, 2013a; Gonnella et al., 2016). The archaeal 16S rRNA tags were primarily associated with different subgroups of the Marine group I *Thaumarchaeota* (84%) (Figure 4A).

### Microbial Community Compositions of the Incubation Experiments

At the end of the Haungaroa sediment slurry incubation experiments (where hydrogen, iron(II) or sulfide were supplemented; see Figure 1 for experimental set-up) the active parts of the microbial communities – based on 16S rRNA gene tags from RNA – were different to those initially detected in the respective natural habitat. Among the potential autotrophs this affected primarily members of *Sulfurovum* and *Sulfurimonas*. *Sulfurovum* were the prevailing Bacteria in the naturally occurring sediments and in most incubations (except for the hydrogen-amended ones) remained dominant, although their proportions generally dropped – from 62% in the sediments to 13, 58, and 35% in the hydrogen-, iron(II)-, and sulfide-spiked experiments, respectively (Figure 3B). In contrast, *Sulfurimonas* increased from 1.5% in the sediment sample to 14% (9-fold), 7% (5-fold), and 24% (16-fold) in the hydrogen-, iron(II)-, and sulfide-spiked incubations, respectively.

**FIGURE 5:**
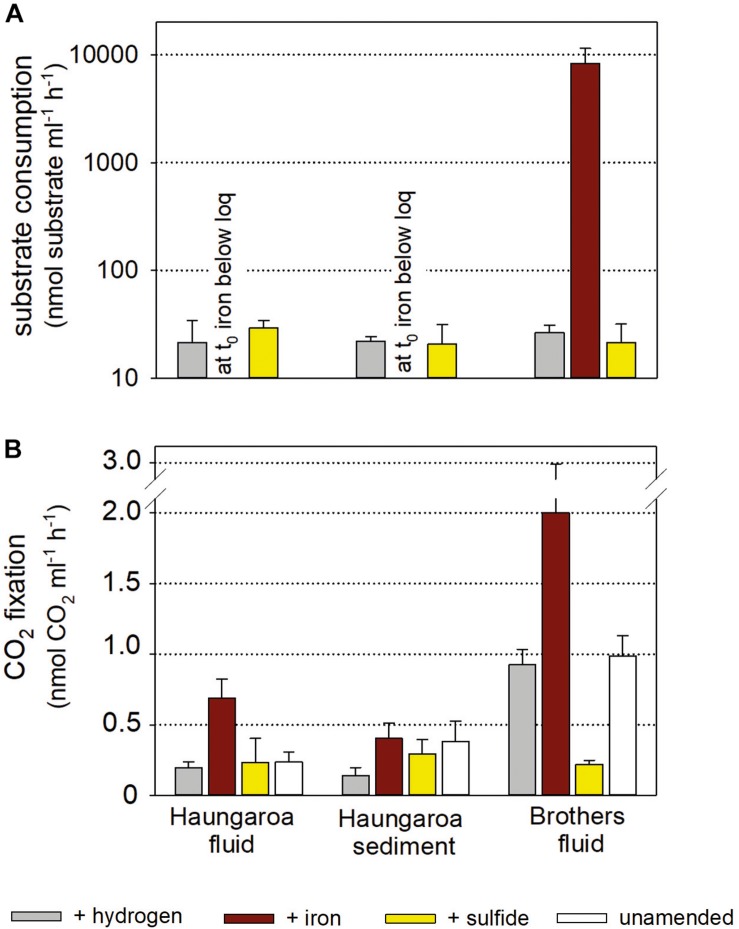
Substrate consumption **(A)** and CO_2_ fixation per volume **(B)** for experiments with Kermadec hydrothermal fluids (30 ROV 4F) and sediment (35 ROV 15M) from Haungaroa and fluids from Brothers (61 ROV 13F-15F). The differently colored bars denote whether the incubated fluid remained unamended (white), supplemented with sulfide (yellow), hydrogen (gray), or iron(II) (brown).

Previous work has proposed that *Sulfurimonas* appear to be better adapted to liquid environments than *Sulfurovum* and that *Sulfurovum* correlates positively with increased oxygen levels (Meier et al., 2017). Hence, the increased numbers of some members of the community in the sediment-slurry experiments (more liquid environment and less oxygen, since incubations were sparged with nitrogen) may thus, be related to experimental conditions. Then, however, one would generally expect a decline in *Sulfurovum* and an increase in *Sulfurimonas* numbers in all sediment-slurry incubations, but this is not the case. For example, in the iron(II)-spiked incubations *Sulfurovum* abundances hardly decreased (from 62 to 58%; Figure 3B), still making up over half of the active community. Likewise, in the sulfide-amended incubations they still comprised more than one third of the bacterial community (35%), while *Sulfurimonas* constituted only one quarter of the bacterial 16S rRNA tags. Consequently, *Sulfurovum’s* predominance in the iron(II)- and sulfide-supplemented enrichments is likely associated with their ability to consume available substrates. However, while monitored sulfide consumption supports this hypothesis, iron was already below its detection limit at the start of the experiment (Figure 5A, see further discussion below in section “Consumption Rates and Autotrophic CO_2_ Fixation in Incubation Experiments”).

*Sulfurovum* have been described as strictly chemolithoautotrophic, metabolically versatile aerobic or anaerobic, sulfur- and hydrogen-oxidizing *Epsilonproteobacteria* (Nakagawa et al., 2007; Mino et al., 2014; Giovannelli et al., 2016). Although genes for iron-uptake are present on the *Sulfurovum* sp. NBC37-1 genome (Nakagawa et al., 2007), no evidence exists supporting *Sulfurovum’s* ability to gain energy by oxidizing iron, neither from experimental approaches nor from known genome sequences (Inagaki et al., 2004; Nakagawa et al., 2007; Park et al., 2012; Mino et al., 2014; Giovannelli et al., 2016; Jeon et al., 2017). Nevertheless, since on different *Sulfurovum* spp.’s genomes, the proportion of “unknown” proteins range from 27 to 30%, one cannot rule out the possibility that proteins associated with iron oxidation are masked among these “unknown” proteins. Consequently, under certain conditions *Sulfurovum* species may be able to oxidize iron, but the general mechanism of microbial iron oxidation is still not uncovered and it is still unknown whether, or to which extent this process is enzymatically catalyzed (Laufer et al., 2016b).

In the Haungaroa hydrogen- and iron(II)-spiked sediment incubation experiments no archaeal amplicons could be retrieved from the transcriptome (Figure 4B and Supplementary Table 2) implying that the experimental conditions are not supportive of the archaeal metabolisms. Nevertheless, considerable archaeal changes were observed in the sulfide-amended incubations. Here, the archaeal diversity decreased rapidly (Supplementary Table 3). 16S rRNA gene tags were related to *Pacearchaeota* and increased 7-fold (to 50%), while the proportion of 16S archaeal rRNA tags related to low abundance and unassigned organisms halved from 81 to 40% (Figure 4B). Culturing Archaea at the laboratory scale has been shown before to be challenging (optimizations with respect to electron donors, pH, temperature, and carbon source) (Lloyd et al., 2005). However, the increase of *Pacearchaeota* in the sulfide-spiked incubations indicates that experimental conditions are beneficial for their growth, albeit sulfur-associated metabolism has not been shown for these Archaea (Castelle et al., 2015).

The differences between the hydrogen-amended Haungaroa fluid incubations and the natural environment were largely associated with low abundance/unassigned Bacteria and Archaea (Figures 3B, 4B). For the other Haungaroa fluid incubations no Archaea could be identified and in several experiments a massive increase of *Actinobacteria* was observed, but no typical chemosynthetic Bacteria were found. The *Actinobacteria* may be a result of contamination.

During Brothers fluid incubation experiments we only observed changes in the bacterial communities (Figure 3B). Almost no archaeal sequences could be recovered from any of the incubations (Figure 4B and Supplementary Table 2) and may indicate that vital nutrients are missing. *Hydrogenovibrio/Thiomicrospira* outcompeted *Sulfurimonas* species in most experiments. The proportion of *Sulfurimonas* 16S rRNA gene tags dropped from 49% in the natural fluid to 9, 15, and 11% in the hydrogen-, iron(II)-, and sulfide-spiked experiments, respectively. Hydrogen- and iron(II)-spiked incubations with Brothers fluid exhibited clear shifts of 16S rRNA gene tags toward active *Hydrogenovibrio*/*Thiomicrospira* species (from 15% in the natural fluids to 52 and 53% in the incubations, respectively).

For more than 40 years, members of the (originally classified as) *Thiomicrospira* group have been described as Bacteria metabolizing sulfur compounds for generating energy chemosynthetically, but recent studies have expanded their metabolic repertoire by, e.g., hydrogen conversion (Hansen and Perner, 2015) and iron cycling (Barco et al., 2017). It is thus not surprising that *Thiomicrospira* species thrive in our iron(II)-spiked incubation experiments, outcompeting other Bacteria like *Sulfurimonas* species. Interestingly, hardly any active *Hydrogenovibrio*/*Thiomicrospira* (<0.01%) were identified in the sulfide-amended incubations. Instead, in these experiments *Sulfurovum* and *Sulfurimonas* were among the prevailing active autotrophic Bacteria (16 and 11%, respectively). Cultured representatives of the genera *Hydrogenovibrio*/*Thiomicrospira* have been shown to grow at sulfide concentrations between 0.01 and 800 μM (Ruby and Jannasch, 1982; Jannasch et al., 1985; Brinkhoff et al., 1999; Takai et al., 2004). Sulfide concentrations in the sulfide-spiked incubation experiment were, with 1 mM sulfide concentration, up to 20% above that range. At least for *Hydrogenovibrio crunogenus*, which is one of the high-sulfide tolerant representatives within the genera *Hydrogenovibrio*/*Thiomicrospira*, it was shown that no CO_2_ was incorporated into biomass when sulfide concentrations exceeded 800 μM (Jannasch et al., 1985). Selected *Sulfurimonas* species, in contrast, were shown to grow at sulfide concentrations up to 3 000 μM (Inagaki et al., 2003b; Takai et al., 2006). Although sulfide tolerances of *Sulfurovum* species have not been specified to our knowledge, our results suggest that prevailing *Sulfurovum* and *Sulfurimonas* species are better adapted to high sulfide contents than *Hydrogenovibrio*/*Thiomicrospira*. One further decisive factor might be the oxygen concentrations, which has been shown to be critical when *Hydrogenovibrio*/*Thiomicrospira* species grow with sulfide as electron donor (Brinkhoff and Muyzer, 1997). The expected low oxygen level in the incubation experiments are thus likely more challenging for microaerobic to facultatively aerobic *Hydrogenovibrio*/*Thiomicrospira* species (Campbell et al., 2006; Boden et al., 2017) than for facultatively anaerobic to microaerobic *Sulfurovum* and *Sulfurimonas* species (Takai et al., 2006; Hügler and Sievert, 2011; Jeon et al., 2017) if sulfide oxidation is the main energy source.

### Consumption Rates and Autotrophic CO_2_ Fixation in Incubation Experiments

Consumption rates monitored in Haungaroa fluid incubations and sediment slurries were 22 ± 12 and 22 ± 2 nmol H_2_ ml^–1^ h^–1^ and 29 ± 5 and 21 ± 10 nmol H_2_S ml^–1^ h^–1^, respectively (Figure 5A). In both sets of experiments, at the start of the experiment iron levels were already below the detection limit (i.e., immediately after iron(II) addition) (Figure 5A) suggesting chemical cross reactions (abiotic oxidation and/or precipitation) so that no iron(II) was likely available for microbial use. The level of autotrophic CO_2_ fixation ranged from 0.2 ± 0.04 and 0.14 ± 0.06 nmol CO_2_ ml^–1^ h^–1^ (hydrogen-amended incubations) over 0.2 ± 0.1 and 0.3 ± 0.1 nmol CO_2_ ml^–1^ h^–1^ (sulfide-amended incubations) to 0.7 ± 0.1 and 0.4 ± 0.1 nmol CO_2_ ml^–1^ h^–1^ (iron(II)-amended incubations) in fluid incubations and sediment slurries, respectively (Figure 5B). Since no iron could be determined in either of the two iron-incubation Haungaroa set-ups at *t*_0_, the measured autotrophic CO_2_ fixation is likely related to other alternative energy sources. Residual sulfide may be present, stimulating biomass synthesis.

The consumption of hydrogen, iron(II) and sulfide in Brothers fluid incubations was 27 ± 4 nmol H_2_ ml^–1^ h^–1^, 26 ± 21 nmol iron ml^–1^ h^–1^, and 21 ± 10 nmol H_2_S ml^–1^ h^–1^, respectively (Figure 5A). Respective autotrophic CO_2_ fixation was 0.93 ± 0.1 nmol CO_2_ ml^–1^ h^–1^ for hydrogen-amended, 2.00 ± 0.9 nmol CO_2_ ml^–1^ h^–1^ for iron(II)-supplemented and 0.22 ± 0.03 nmol CO_2_ ml^–1^ h^–1^ for sulfide-spiked incubations (Figure 5B). Both the consumption rates and the CO_2_ fixation rates were comparable to values previously measured in hydrothermally influenced habitats along the MAR (Perner et al., 2013b and references therein; Adam and Perner, 2018). So far, we are not aware of any similar incubation experiments with hydrothermal samples that have been set-up with iron(II) as a substrate. However, experiments with coastal marine sediments incubated with iron(II) showed that iron(II) consumption and respective autotrophic CO_2_ fixation (Laufer et al., 2016a, b) was consistent with what was found in our hydrothermal Brothers fluid incubations.

### Gibbs Energy and Consumption to CO_2_ Fixation Ratios

Based on the empirical Gibbs energy dissipation coefficient a relation exists between the oxidation of electron donors (catabolism) and the fixation of CO_2_ (anabolism) (Heijnen and Vandijken, 1992): 1060 kJ are required for hydrogentrophy (no reverse electron transport is required) and 3500 kJ for sulfide and iron(II) oxidation (reverse electron transport is required) to fix 1 mol of carbon into biomass. Given the values of Δ_r_G of the different catabolic reactions (see Table 3) the estimated molar ratio of electron donor consumption over the amount of CO_2_ fixed can vary considerably with 4:1, 4:1, and 35:1 for aerobic sulfide, hydrogen, and iron oxidation, respectively, as well as 8:1, 4:1, and 40:1 for anaerobic sulfide, hydrogen, and iron oxidation, respectively (see Table 3).

**TABLE 3:** Chemical reactions and standard Gibbs energies per mol of electron donor for different catabolic reactions.

**Chemical reaction**	**Δ_*r*_G′^0^ (kJ per mol reaction)**	**Δ_*r*_G′^0^ (kJ per mol e-donor)**	**Ratio^∗^ Sb:CO_2_**
**Sulfide oxidation**			
H_2_S(aq) + 2O_2_(aq)→ SO_4_^2–^ + 2H^+^	−829.6	−829.6	4.2
H_2_S(aq) + 4NO_3_^–^ → SO_4_^2–^ + 4NO_2_^–^ + 2H^+^	−440.2	−440.2	8.0
**Hydrogen oxidation**			
2H_2_(aq) + O_2_(aq)→ 2H_2_O	−526.2	−263.1	4.0
5H_2_(aq) + 2NO_3_^–^ + 2H^+^ → N_2_(aq) + 6H_2_O	−1191.5	−238.8	4.4
**Iron oxidation**			
10Fe^2+^ + 2NO_3_^–^ + 24H_2_O → 10Fe(OH)_3_(s) + N_2_ + 18H^+^	−880.1	−88.0	39.8
Fe^2+^ + 2½H_2_O + 1/2_2_(aq) → Fe(OH)_3_(s) + 2H^+^	−100.4	−100.4	34.9

*Δ_*r*_G′^0^ is standard the Gibbs energy of reaction for 10^5^ Pa, 298K, and a pH of 7 calculated from Geochemist’s Workbench (Bethke, 2007) using the thermo. dat database. Sb:CO_2_, Substrate consumption to CO_2_ fixation; ^∗^to fix 1 mol of carbon into biomass 1060 kJ are required if no reverse electron flow is needed (H_2_ oxidation) and 3500 kJ if a reverse electron flow occurs (iron(II) oxidation and sulfide oxidation) (Heijnen and Vandijken, 1992).*

In the Haungaroa sediment incubations the calculated ratio of H_2_:CO_2_ 157:1 suggests that 2.6% and 2.8% of the energy provided through oxic and anoxic hydrogen oxidation, respectively, is going into biomass synthesis, if all microorganisms in the experiments were catalyzing this reaction. Depending on whether sulfide oxidation is coupled to oxygen or nitrate reduction, the H_2_S:CO_2_ ratio of 70:1 indicates that 6.0% (oxic) or 11.4% (anoxic) of the energy gained through sulfide oxidation could be used for CO_2_ fixation under the provided conditions, if all organisms in the experiments are catalyzing the same chemical reactions. Ratios of hydrogen to fixed CO_2_ indicate a minor role of hydrogen consumption for biomass synthesis in this incubation. Hence, sulfide oxidation appears to be the prevailing energy source for biomass synthesis. The experimental data are therefore in line with the thermodynamic predictions for the environment (Figure 2).

These energy requirements for autotrophic CO_2_ fixation are typically estimated on the hexose level via the Calvin–Benson–Bassham (CBB) cycle (Stouthamer, 1973; Kelly, 1982; Heijnen and Vandijken, 1992). However, the reductive tricarboxylic acid (rTCA) cycle, an alternative autotrophic CO_2_ fixation pathway, has been demonstrated to operate in all autotrophic *Epsilonproteobacteria* tested so far, including *Sulfurovum* species (Nakagawa et al., 2007; Han and Perner, 2015) that were abundant in the investigated Haungaroa sediments and experiments. While it has been estimated that the CBB cycle requires on average 0.238 moles ATP to synthesize one gram biomass, the rTCA cycle was calculated to need average of only 0.195 moles ATP for the synthesis of one gram biomass (Mangiapia and Scott, 2016). Hence, if the rTCA cycle was used as a base for estimating our substrate consumption to CO_2_ fixation ratios, the relative amount of energy that was fed into CO_2_ fixation would decrease to roughly 56% (corresponding to 2.1 and 4.9% for aerobic hydrogen and sulfide oxidation, respectively, and 2.3 and 9.3% for anaerobic hydrogen and sulfide oxidation, respectively). Thus, even if the rTCA cycle was used, sulfide oxidation supports the largest amounts of biomass synthesis in these experiments under the provided conditions.

The ratios of 109:1 for H_2_:CO_2_ and 124:1 for H_2_S:CO_2_ in Haungaroa fluid incubations indicate that these inorganic electron donors are not fueling large amounts of primary production (3.7 and 4.1% under oxic and anoxic, respectively, hydrogen-amended conditions; 3.4 and 6.4% under oxic and anoxic, respectively, H_2_S-amended conditions). Given that in the incubation experiments the dominant classified Bacteria were all common organotrophs, the little observed biomass synthesis is likely related to phylogenetically diverse organisms summarized as <5% (unknown) rest (Figures 3B, 4B).

In the different Brothers fluid experimental incubations the consumption to CO_2_ fixation ratios varied considerably. The H_2_:CO_2_ fixation ratio was 28:1 implying that theoretically about 14.4 or 15.9% of the gained energy from hydrogen oxidation under aerobic or anaerobic conditions, respectively, was used for CO_2_ fixation in the experiments – if all organisms were consuming hydrogen. The iron to CO_2_ fixation ratio of 13:1 suggests that all of the energy gained through iron(II) oxidation is channeled to autotrophic CO_2_ fixation and other oxidation processes are additionally fueling biomass synthesis. H_2_S:CO_2_ fixation ratios of 97:1 propose that about 4.3% or 8.2% under aerobic or anaerobic conditions, respectively, of the sulfide oxidation derived energy is fueling autotrophic CO_2_ fixation. As already mentioned above, the energy requirement for CO_2_ fixation is typically estimated based on the energy cost intensive CBB cycle [0.238 moles ATP per g biomass (Mangiapia and Scott, 2016)]. If thus the lower-energy requiring rTCA cycle [0.195 moles ATP per g biomass (Mangiapia and Scott, 2016)] would be used as a base for calculating substrate consumption to CO_2_ fixation ratios, the theoretical amount of energy that were fed into CO_2_ fixation would decrease to 11.8 and 3.6% for aerobic hydrogen and sulfide oxidation, respectively, and 13.0 and 6.7% for anoxic hydrogen and sulfide oxidation. Still, all of the energy gained through iron(II) oxidation would be expected to fuel autotrophic CO_2_ fixation. Conclusively, in these experiments iron(II) and hydrogen support most of the biomass synthesis but also sulfide oxidation can support autotrophic CO_2_ fixation under anoxic conditions considerably. This is in line with the catabolic energy estimates. Overall the CO_2_ fixation rates are too low to cover the whole inorganic electron donor consumption. This might indicate that chemolithoheterotrophs are active and consume the remaining inorganic electron donors for their heterotrophic growth. *In vivo* autotrophic and heterotrophic community members thus likely compete against available electron donors. However, possible autotroph-heterotroph interactions could just as well strengthen the microbial communities (i.e., improve productivity, stability, and robustness) like it has been shown before for a Fe(III)-oxide mat microbial community of Yellowstone National Park (Hunt et al., 2018).

### Key Players for Element Cycling and Primary Production in Haungaroa Sediment and Brothers Fluid

From all experiments those with Brothers fluids amended with iron(II) exhibited the largest autotrophic CO_2_ fixation potential. Hydrogen and to some extend also sulfide also stimulated biomass synthesis in the Brothers fluid incubations agreeing with catabolic estimates. *Hydrogenovibrio*/*Thiomicrospira* were highly abundant under most experimental conditions. In the sulfide-spiked incubations *Sulfurovum* were among the prevailing Bacteria suggesting that *Sulfurovum* species are better adapted to higher sulfide concentrations, while the growth of presented *Hydrogenovibrio*/*Thiomicrospira* species seems to be negatively affected if sulfide concentrations are ≥1 mM. The Haungaroa fluids, in contrast, indicated considerably less primary production based on the above mentioned inorganic electron donors under the provided conditions. No abundant common autotrophic microorganisms could be identified after the incubations experiments that were likely responsible for the CO_2_ fixation. Autotrophic CO_2_ fixation was the highest in the Haungaroa sediment experiments when based on sulfide oxidation. *Sulfurovum* species appeared to be best adapted to these experimental conditions matching the physiology with respect to being able to consume sulfide and fix CO_2_ autotrophically and coincides with the catabolic energy estimates for the environment.

Although phenotypes of *Sulfurimonas*, *Sulfurovum*, and *Hydrogenovibrio*/*Thiomicrospira* are very similar at the first glance, representatives differ from each other in small details (e.g., oxygen requirements, sulfide tolerances, etc.). Similarities include that they grow at moderate temperatures, can live autotrophically, and can consume hydrogen or reduced sulfur compounds for energy generation. However, *Hydrogenovibrio*/*Thiomicrospira* use the CBB cycle with a high energy requirement, whereas *Sulfurimonas* and *Sulfurovum* operate the rTCA cycle, with a low energy requirement. Also the *Epsilonproteobacteria* are better adapted to low oxygen levels or oxygen absence, while *Hydrogenovibrio*/*Thiomicrospira* are limited to environments where oxygen is available. Our data support that species of each genera, in their own specific manner, are perfectly adapted to dynamic hydrothermally influenced environments. We thus assume that *Sulfurimonas*, *Sulfurovum*, and *Hydrogenovibrio*/*Thiomicrospira* species present a chemically fine-tuned vent community of primary producers in the Haungaroa sediments and Brothers fluids.

## Experimental Procedures

### Sample Collection

Two hydrothermal fluids and one sediment sample were collected from two Kermadec arc locations with the remotely operated vehicle ROV Quest (MARUM, University of Bremen) during the HYDROTHERMADEC cruise (SO253, December 2016/January 2017) with the RV Sonne. The hydrothermal fluid samples were retrieved with the pumped flow-through system KIPS (Kiel Pumping System) (Garbe-Schönberg et al., 2006) from a Brothers caldera wall location (061 ROV 13F-15F; water depth 1643 m; 34°51.73′S; 179°03.45′E) and from the Haungaroa fluid site (30 ROV 4F), water depth 674 m, 32° 36.95′S; 179° 37.39′W (for sampling sites see Supplementary Figure 1). For further details on sampling with the KIPS see Perner et al. (2009). The sediment sample was collected from a Haungaroa site (35 ROV 15M; water depth 677 m; 32° 36. 95′S; 179° 37.48′W) with a shovel (for sampling site see Supplementary Figure 1, for illustration of sampling tools see Supplementary Figure 2). The distance between the two working areas is about 280 km. Both samples were immediately processed after retrieval onboard.

### Sediment Characterization

Bulk sample chemistry was determined at the ICBM Institute for Chemistry and Biology of the Marine Environment in Oldenburg. The ground and dried sample was fused in a platinum crucible to glass beads with lithium tetraborate and ammonium nitrate. X-ray fluorescence analyses were carried out on a wavelength-dispersive XRF spectrometer (Axios plus device from Panalytical). The device was calibrated with *n* = 66 samples and trueness and precision was monitored by measurements of the international standard NOD-A-1 (Flanagan and Gottfried, 1980) and two in house standards, a black shale and a basalt. Trueness and precision of the measurement was better than 5% for major elements and better than 15% for trace elements. Sulfur was determined via combustion analyses (ELTRA CS Analyzer) with the same precision as for the other major elements (better than 5%).

### Incubation Experiments

The hydrothermal fluid samples and the sediment sample were used for incubation experiments (for details on set-up see Figure 1) to determine consumption of hydrogen, sulfide, and iron(II) as well as respective CO_2_ fixation (endpoint measurements) and the microbial community composition. Incubation experiments were set-up within 2 h of sample recovery. Detailed descriptions of sample preparation procedures can be found in Perner et al. (2010, 2011). In short, four sets of experiments (each in six parallels) were performed. For the fluid sample 4 × 6 hungate tubes were filled with 10 ml of undiluted fluids. For the sediment sample 2 ml sediment were transferred in each of the 4 × 6 hungate tubes filled with 10 ml artificial seawater (Sako et al., 1996). All incubations were set under a N_2_ atmosphere to avoid oxidation. From the four sets (i) one set was spiked with a 1:40 dilution of a hydrogen mix [125 μl of a 80% H_2_: 20% N_2_ purity 5.0 (Westfalen AG) injected in 5 ml N_2_ headspace ? 700 μM H_2_ in headspace or 12–14 μM H_2_ in solution], (ii) one set was supplemented with sodium sulfide (1 mM end-concentration), (iii) one set was amended with iron(II) (0.4 mM endconcentration), and (iv) one set was kept unamended as a control. Of each set (six subsamples), three were supplemented with radioactively labeled ^14^C-bicarbonate (Hartmann Analytic, Braunschweig, Germany) (final activity 30 kBq mL^–1^) and the other three were amended with unlabeled 15 nM sodium bicarbonate and used for consumption measurements and cell material concentrations. All incubations were allowed to grow for 8 h at 18°C in the dark. After incubation, formaldehyde was added to the samples (final concentration: 2%) and samples were subsequently left undisturbed for a further 12 h at 4°C to stop microbial activity. To prepare samples for biologically incorporated inorganic carbon determination the three parallels of each set amended with radioactively labeled bicarbonate were concentrated on filters (0.2 μm GTTP, Merck Millipore Ltd., Darmstadt, Germany). Excess CO_2_ was removed by acidification and aeration. Carbon incorporation measurements were performed on-board using a scintillation counter (Perkin Elmer, Waltham, MA, United States). For calculating CO_2_ fixation, the measured DIC values of 2.38 mM for Haungaroa and 2.87 mM for Brothers (H. Strauss, Univ. Münster; Germany, unpublished data) were used. At the start and at the end of the incubation experiments (in samples with unlabeled bicarbonate) hydrogen, iron and sulfide were measured as described in the fluid chemistry section. The remaining sample was used for concentration of cell material on polycarbonate filters (0.2 μm, GTTP, Merck Millipore Ltd.) and was immediately stored at −80°C for later RNA analyses of 16S rRNA tags (see below). To exclude that substrate consumption or CO_2_ fixation was related to abiogenic processes or reactions, control experiments were performed with filtered (pore size 0.1 μm), and autoclaved hydrothermal fluid.

### Chemistry

Consumption of hydrogen, iron and sulfide was measured from the incubations at the beginning and at the end of the experiments. Hydrogen concentrations were determined by gas chromatography with a 7820A Agilent gas chromatograph (Agilent Technologies, Santa Clara, CA, United States) that was configured with a Molecular Sieve 5A 60/80 μm column (Sigma-Aldrich, St. Louis, MO, United States) and a thermal conductivity detector (Agilent Technologies, Santa Clara, CA, United States), using nitrogen as carrier gas. Sub samples from the incubation experiment were transferred into gas-tight glass syringes and a headspace extraction was subsequently executed. Hydrogen concentrations of the subsamples were then referred to the sample volume of the incubation experiments. The gas chromatograph was calibrated using reference gases with 1.031 mol% H_2_ and 253 mol% H_2_ in a matrix of nitrogen (Crystal-Mix, Air Liquide).

Spectral photometry of dissolved sulfide was performed following the methylene blue method (Cline, 1969). Total dissolved iron concentrations were measured at the Trace Element Centre of the University of Otago (New Zealand) using an Agilent 7500ce Quadrupole Inductively Coupled Plasma-Mass Spectrometer (q-ICP-MS) equipped with an octopole collision cell and an autosampler. For detailed information on sample preparation and operating conditions of the instrument see Talbot and Weiss (1994) and Wolf and Adams (2015). In order to assess possible matrix effects and instrumental drifts during the run, samples were spiked off-line with an internal multi-element standard (Linge and Jarvis, 2009; Wolf and Adams, 2015). External calibration standards for the q-ICP-MS analysis were prepared via a serial dilution of a commercially available SPEX CertiPrep multi-element standard (NIST traceable) in 2% v/v quartz distilled HNO_3_. For quality control, method blanks (Milli-Q*; n* = 3), spiked samples (*n* = 5), and a low metal reference seawater sample spiked with 35.81 nM iron (*n* = 6) were periodically analyzed throughout the run. Obtained data was dilution- and blank-corrected to receive accurate analyte levels in the undiluted sample. The procedural blanks showed no iron contamination (below detection limit of 2.7 nM) and the precision of the multiple spiked samples (±0.3%) was within a valid range. The accuracy (measured reference seawater value: 35.72 ± 0.81) of the method was acceptable (2.26% SD).

### DNA and RNA Extraction, cDNA Generation, Amplification of 16S Tags, and Sequencing

DNA was extracted from half of the filters of the hydrothermal fluid sample (Brothers) and from 400 mg of the sediment sample (Haungaroa) using the DNeasy PowerSoil Kit (Quiagen, Venlo, Netherlands) according to manufacturer’s instructions. RNA was extracted from half of the filters of all previously mentioned incubation experiment samples using the Direct-zol^TM^ RNA Miniprep Plus Kit (Zymo, Irvine, CA, United States) according to manufacturer’s instructions followed by a second DNaseI digestion step by using the DNase Max kit (Quiagen), following the provided protocol. Isolated RNA was used to synthesize total cDNA with Invitrogen’s SuperScript^®^ VILO^TM^ cDNA Synthesis Kit (Life Technologies^TM^, Darmstadt, Germany), according to manufacturer’s instructions.

The isolated DNA and the generated cDNA served as a template (5 ng) for paired-end 16S rRNA gene sequencing on an Illumina MiSeq platform like it has been described before (Gonnella et al., 2016). In short, the hypervariable V3 and V4 regions of the bacterial and the hypervariable V4 and V5 regions of the archaeal 16S rRNA gene were amplified with specific amplicon primer sets. The primer set that was used for the amplification of the bacterial hypervariable V3 and V4 regions was S-D-Bac-0341-b-S-17 (forward primer 5′-CCT ACG GGN GGC WGC AG-3′) and S-D-Bac-0785-a-A-21 (reverse primer 5′-GAC TAC HVG GGT ATC TAA TCC-3′) (Klindworth et al., 2013). For the amplification of the archaeal hypervariable V4 and V5 regions we decided to use two different primer sets in order to increase the coverage: (i) Arch_519F (forward primer 5′-CAG YMG CCR CGG KAA HAC C-3′) and Arch 915R (5′-RGT GCYC CCC CGC CAA TTC-3′) (Ding et al., 2017) and (ii) Arch 524F (forward primer 5′-TGY CAG CCG CCG CGG TAA-3′) and Arch 958R (reverse primer 5′-CCG GCG TTG AVT CCA ATT-3′) (Cerqueira et al., 2017). Each primer contained an Illumina^®^ adaptor overhang nucleotide sequence next to the broadly conserved primer sequence (forward primer adapter: 5′-TCG TCG GCA GCG TCA GAT GTG TAT AAG AGA CAG-3′ and reverse primer adapter: 5′-GTC TCG TGG GCT CGG AGA TGT GTA TAA GAG ACA G-3′). For each sample two separate PCRs were performed for each of the three primer sets (in total 2 × 3 PCRs per sample) with the Kapa Hifi HotStart Ready Mix (Kapa Biosystems, Boston, MA, United States) according to manufacturer’s instructions under the following PCR conditions: 3 min initial denaturation followed by 25 cycles of denaturation at 95°C for 30 s, primer annealing at 55°C (for amplification of the bacterial 16S rRNA gene) or at 63°C (for amplification of the archaeal 16S rRNA gene) for 30 s and extension at 72°C for 30 s. The 1 × 2 parallel samples of the bacterial 16s rRNA gene amplification and the 2 × 2 parallel samples of the archaeal 16S rRNA amplification were pooled and purified by using Agencourt AMPure XP beads (Beckman Coulter, Brea, CA, United States) according to manufacturer’s protocol. In a subsequent amplification step multiplexing indices and Illumina^®^ sequencing adapters were added to the pooled and purified amplicons by using the Nextera^®^ XT Index Kit (Illumina^®^, San Diego, CA, United States) according to manufacturer’s instructions. The Index PCR was again performed with the Kapa Hifi HotStart Ready Mix (Kapa Biosystems) as described above but with only 8 cycles. Prior to quantification of the final library the indexed samples were purified by using Agencourt AMPure XP beads (Beckman Coulter) according to manufacturer’s protocol. Concentrations of all 2 × 10 purified samples were estimated with a NanoDrop2000 spectrophotometer (ThermoFisher Scientific, Waltham, MA, United States). The measured DNA concentrations were between 5 and 77 ng μl^–1^. The purified PCR product samples were pooled in a way that all subsamples contained an equimolar amount of 16S rRNA gene amplificate. This library pool was then analyzed by the 2100 Bioanalyzer (Agilent Technologies, using the DNA High Sensitivity Chip). The amplicon libraries were sequenced by paired-end sequencing in a 2 × 300 bp run on the MiSeq platform (Illumina, St. Diego, United States).

### Analyses of 16S Tags

The demultiplexed forward and reverse reads were joined using Flash v.1.2.11 (Magoc and Salzberg, 2011) with default parameters and eliminated non-merging read pairs. The resulting amplicon sequences were filtered by quality, trimmed and imported in Qiime v.1.9 (Caporaso et al., 2010) using split_libraries_fastq. py with default parameters. Quality control on the raw and preprocessed reads was performed using Fastqc v.0.11.5.^1^ Operational taxonomic unit (OTU) picking was performed using the open-reference procedure (Rideout et al., 2014), as implemented in Qiime, based on UCLUST v.1.2.22q (Edgar, 2010), and Silva v.119 (Quast et al., 2013). The OTU identity threshold was 97% and singleton OTUs (single sequence in the whole dataset) were removed. Taxonomy labels were assigned to the OTUs by Qiime (pick_open_reference_otus. py script). Spurious assignments outside of Bacteria (for the amplification with bacterial primers) or Archaea (for the amplification with archaeal primers) were removed (i.e., the corresponding counts were moved to unassigned).

1http://www.bioinformatics.babraham.ac.uk/projects/fastqc/

## Data Availability Statement

Sequence data was deposited at the Sequence Read Archive (SRA) of the National Center for Biotechnology Information (NCBI) under the BioProject PRJNA525429.

## Author Contributions

SB performed DNA and RNA extraction, cDNA generation, and amplification of 16S rRNA gene tags. KS set up incubation experiments and measured CO_2_ fixation. GG analyzed microbial 16S rRNA gene tags with the support from SK. AD and WB measured hydrogen consumption and performed catabolic energy estimates. CK, RZ, AK, and SS measured iron. DI performed MiSeq sequencing. MP interpreted the obtained data with input from SB, WB, and CK and wrote the manuscript with major contributions from SB, WB, and CK and approval of all authors.

## Conflict of Interest

The authors declare that the research was conducted in the absence of any commercial or financial relationships that could be construed as a potential conflict of interest.

## Footnotes

1 http://www.bacterio.net
