# Supplementary material for: Parameters Governing the Community Structure and Element Turnover in Kermadec Volcanic Ash and Hydrothermal Fluids as Monitored by Inorganic Electron Donor Consumption, Autotrophic CO2 Fixation and 16S Tags of the Transcriptome in Incubation Experiments
Source: Front Microbiol. 2019 Oct 9;10:2296. doi: 10.3389/fmicb.2019.02296 (PMC6794353; doi:10.3389/fmicb.2019.02296)

Supplementary Information for

Parameters governing the community structure and element turnover in Kermadec volcanic ash and hydrothermal fluids as monitored by inorganic electron donor consumption, autotrophic CO_2 fixation and 16S tags of the transcriptome in incubation experiments

Stefanie Böhnke[1,2], Katharina Sass[2], Giorgio Gonnella[3], Alexander Diehl[4], Charlotte Kleint[5], Wolfgang Bach[4], Rebecca Zitoun[6], Sylvia G. Sander[6,7] Andrea Koschinsky[5], Daniela Indenbirken[8], Stefan Kurtz[3] and Mirjam Perner[1,2]*

[1]present address: Helmholtz Centre for Ocean Research Kiel (GEOMAR), Geomicrobiology, Wischhofstr. 1-3, 24148 Kiel

[2]Molecular Biology of Microbial Consortia, University of Hamburg, Biocenter Klein Flottbek, Ohnhorststr. 18, 22609 Hamburg, Germany

[3]Universität Hamburg, MIN-Fakultät, ZBH - Center for Bioinformatics, Bundesstraße 43, 20146 Hamburg, Germany

[4]Department of Geosciences and MARUM – Centre for Marine Environmental Science, University of Bremen, Klagenfurter Str. 2-4, 28359 Bremen, Germany

[5]Department of Physics and Earth Sciences, Jacobs University Bremen, Campus Ring 1, Bremen D-28759, Germany

[6]Department of Chemistry, University of Otago, P.O.Box 56, Dunedin 9054, New Zealand

[7]present address: Marine Environment Studies Laboratory, International Atomic Energy Agency, 4 Quai Antione 1er, 98000, Monaco, Monaco

[8]Heinrich-Pette-Institut, Leibniz Institute for Experimental Virology, Martinistraße 52, 20251 Hamburg, Germany

This file contains:

Supplementary Tables 1-3

Supplementary Figures 1-2

*Corresponding Author: Mirjam Perner. Mailing Address: GEOMAR Helmholtz Center for Ocean Research Kiel, Geomicrobiology, Wischhofstraße 1-3, 24148 Kiel. Tel: +49-431-6002837, Fax: +49-431-600-2941, E-mail: mperner@geomar.de

Supplementary Tables

Supplementary Table 1: Predicted versus observed compositions of diffuse vents.

Chemical compounds	Brothers		Haungaroa	
	predicted	observed	predicted	observed
H_2(aq) µM	0.26	0.126	0.49	0.008
CH_4(aq) µM	0.09	0.092	0.19	0.02
HCO_3^- mM	2.3	2.87	5.1	2.4
H_2S(aq) µM	47	15	44	<1
Fe^{2+} µM	529	179	9.5	<1

Supplementary Table 2: Number of merged bacterial and archaeal reads sequenced from the environment and the different incubation experiments.

sample	bacterial merged reads	archaeal merged reads
**Haungaroa sediment**		
A (environment)	382577	235570
B (+ hydrogen)	190764	-
C (+ iron(II))	231175	-
D (+ sulfide)	466356	21921
E (unamended)	743770	144835
**Brothers fluid**		
A (environment)	436731	625078
B (+ hydrogen)	234579	2012
C (+ iron(II))	354834	-
D (+ sulfide)	64621	-
E (unamended)	103048	-

Supplementary Table 3: Shannon diversity index.

sample	Bacteria	Archaea
**Haungaroa sediment**		
A (environment)	5.3	8.9
B (+ hydrogen)	6.1	-
C (+ iron(II))	5.5	-
D (+ sulfide)	6.4	3.2
E (unamended)	6.4	7.5
**Brothers fluid**		
A (environment)	5.7	4.3
B (+ hydrogen)	3.0	-
C (+ iron(II))	3.1	-
D (+ sulfide)	5.0	-
E (unamended)	4.4	-

Supplementary Figures

Supplementary Figure 1: Map of sampled Haungaroa sediment and Brothers hydrothermal fluid located at the Kermadec arc, New Zealand. The sampling sites are depicted by yellow stars. The map was created using GeoMapApp software version 3.6.8 (www.geomapapp.org).

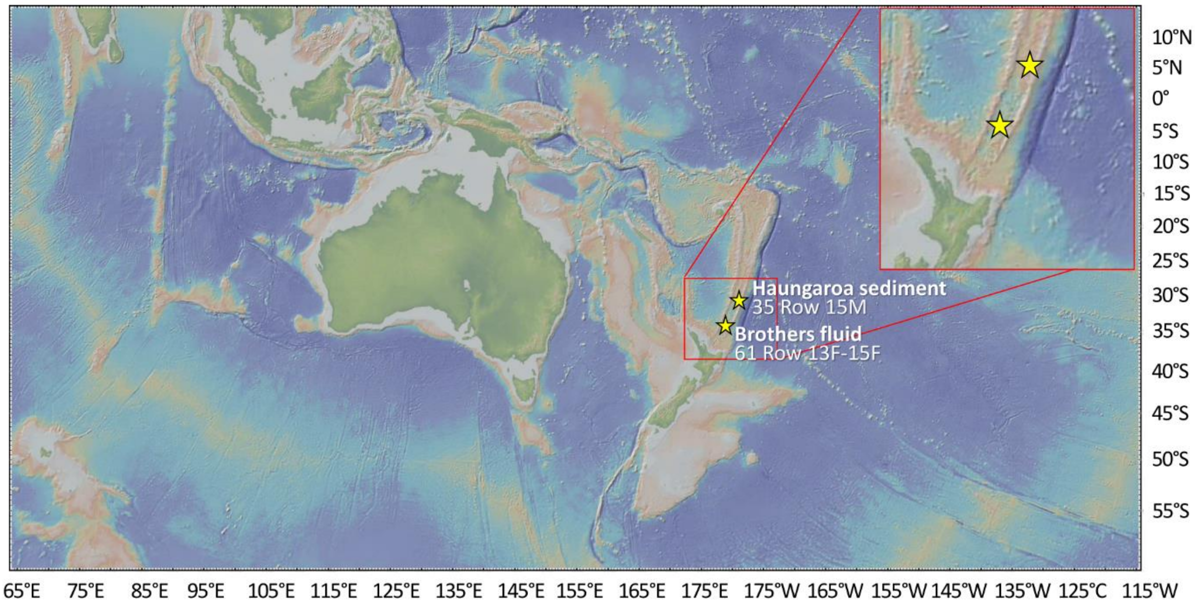

Supplementary Figure 2: (A) Overview of the Haungaroa sediment sampling area and (B) illustration of sampling equipment used at the Haungaroa’ volcano to sample sediment with a shovel. Both pictures show that the prevalent sediment is covered by a white microbial mat. Pictures are protected by copyright of the MARUM (Center of Marine Environmental Sciences, Research Faculty University of Bremen).

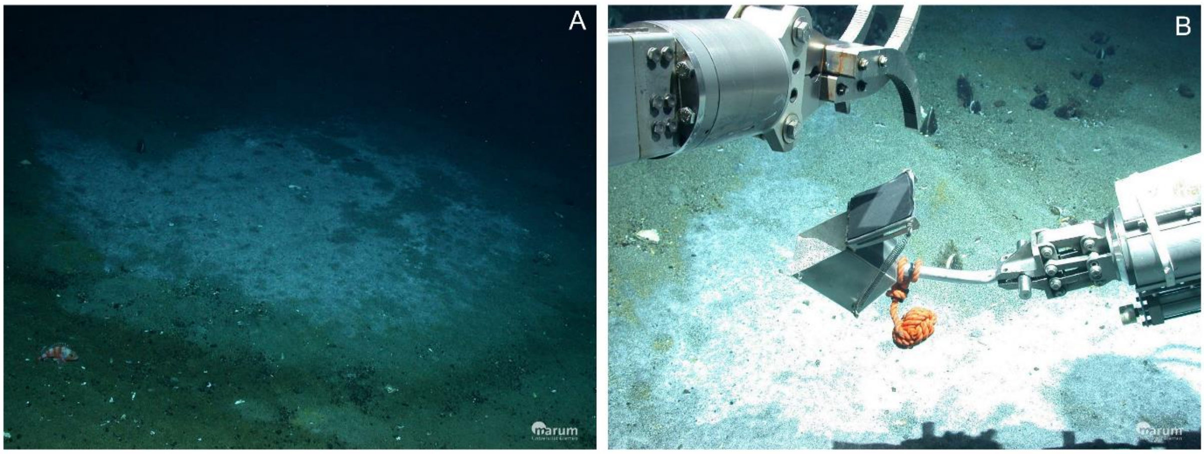

Supplement: Supplementary file 1 [file Data_Sheet_1.pdf]
